# Big Field of View MRI T1w and FLAIR Template - NMRI225

**DOI:** 10.1038/s41597-023-02087-1

**Published:** 2023-04-14

**Authors:** Barbara A. K. Kreilkamp, Pascal Martin, Benjamin Bender, Christian la Fougère, Daniel van de Velden, Christina Stier, Silke Ethofer, Raviteja Kotikalapudi, Justus Marquetand, Erik H. Rauf, Markus Loose, Niels K. Focke

**Affiliations:** 1 Clinic for Neurology, University Medical Center Göttingen, Göttingen, Germany; 2 Department of Neurology and Epileptology, Hertie Institute of Clinical Brain Research, University of Tübingen, Tübingen, Germany; 3 Center for Neuro-Oncology, Comprehensive Cancer Center Tübingen Stuttgart, University Hospital Tübingen, Eberhard-Karls University of Tübingen, Tübingen, Germany; 4 Department of Neuroradiology, University Hospital Tübingen, Eberhard Karls University Tübingen, Tübingen, Germany; 5 Department of Nuclear Medicine, Eberhard Karls University, Tübingen, Germany; 6 Department of Neurosurgery, University of Tübingen, Tübingen, Germany; 7 University of Duisburg-Essen, Essen, Germany; 8 Department of Neural Dynamics and Magnetoencephalography, Hertie Institute of Clinical Brain Research, University of Tübingen, Tübingen, Germany; 9 MEG-Center Tübingen, University of Tübingen, Tübingen, Germany

**Keywords:** Brain, Computational neuroscience

## Abstract

Image templates are a common tool for neuroscience research. Often, they are used for spatial normalization of magnetic resonance imaging (MRI) data, which is a necessary procedure for analyzing brain morphology and function via voxel-based analysis. This allows the researcher to reduce individual shape differences across images and make inferences across multiple subjects. Many templates have a small field-of-view typically focussed on the brain, limiting the use for applications requiring detailed information about other extra-cranial structures in the head and neck area. However, there are several applications where such information is important, for example source reconstruction of electroencephalography (EEG) and/or magnetoencephalography (MEG). We have constructed a new template based on 225 T1w and FLAIR images with a big field-of-view that can serve both as target for across subject spatial normalization as well as a basis to build high-resolution head models. This template is based on and iteratively re-registered to the MNI152 space to provide maximal compatibility with the most commonly used brain MRI template.

## Background & Summary

Brain templates are commonly utilized within the neuroimaging community and are needed for spatial normalization of imaging datasets acquired from different individuals to the same stereotactic space. These templates can be used to either: (a) reduce individual shape and size variations prior to MRI structural/functional analyses or (b) to extract signal from specific brain areas in region-based analyses of structure or function using an atlas^1^. Morphological differences can be significant across individual brains. Consequently, sample size and population differences are key factors influencing brain template construction^2^.

Commonly used templates within the neuroimaging community such as those provided by the Montreal Neurological Institute (MNI) frequently (exceptions are discussed) have a field-of-view (FOV) focused on the brain, i.e. the image space does not cover the full head (often excluding nasion, inion and auricular landmarks). Specifically, MNI has made the MNI305 (9 degrees of freedom linear co-registration of 305 normal MRIs to Talairach space, where translation, rotation and scaling are performed along the three axes x, y, z), Colin27 original (single subject scanned 27 times), MNI152 linear (152 MRIs were linearly co-registered (9 degrees of freedom) to the MNI305 space), MNI152 NLIN (152 MRIs were non-linearly co-registered into MNI305 space), Colin27 hires T1/T2 version (2008) and MNI152 NLIN 2009 (a second nonlinear co-registration of the 152 MRIs, best resolution to date) available (https://www.lead-dbs.org/about-the-mni-spaces/). Additionally, the community has made the ICBM152 extended nonlinear atlas (2020) available to the public (https://nist.mni.mcgill.ca/icbm-152-extended-nonlinear-atlases-2020/). However, this template does not involve a FLAIR version and has a focus on the intracranial structures. The limited spatial coverage of all MNI templates is sufficient for most brain MRI analyses and was useful to save disk space. Here we have used the MNI152 NLIN 6^th^ generation asymmetric variant as a basis for our template generation as this is the most commonly used brain MRI template. We have constructed a new template based on 225 T1w and FLAIR images with a big FOV that can serve both as target for across subject spatial normalization as well as a basis to build high-resolution head models. The following section describes the motivation and exact reasons for the generation of our brain template.

Given these limitations the anatomical detail and coverage of extra-cranial areas is suboptimal. This hinders usage of typical brain templates for applications that require a precise knowledge of these structures. One such application is source reconstruction of electroencephalography (EEG) data that greatly benefits from a high-quality head model. EEG source imaging (ESI or source reconstruction) is a model-based representation technique that integrates temporal and spatial components of EEG to identify the generating source of electrical potentials recorded on the scalp. Kaiboriboon *et al*.^3^ have provided a review on ESI and highlight the value of ESI in pre-surgical evaluation of patients with epilepsy and in precise localization of eloquent cortex. A realistic head model is crucial to solving the ESI algorithms^4–6^. Although less relevant for head modeling, source reconstruction based on magneto-encephalography (MEG) also requires anatomical knowledge of the positioning of the head in the dewar of the system.

Moreover, in source reconstruction based on EEG/MEG it is of pivotal importance to localize certain anatomical landmarks (nasion, pre-auricular points) that are not well covered in the MNI template. Even worse, when using high-density EEG systems, like the EGI/Magstim 256-channel caps, or EEG montages with lower temporal electrodes, like the IFCN2017 array^7^, these include electrode positions that are outside the FOV of conventional templates. Finally, a standardized canonical space template, such as the one provided here, may serve as a standard when an individual MRI is not available for head modeling. This head modeling is impossible to do with current brain-centric templates given they have limited detail and coverage of extra-cranial areas. The template presented here may also enable machine-learning scientists to enhance their studies with insights from the brain’s anatomy and neurophysiology through MEG and EEG^8^. Finally, our template can be used as a face and skull template for other purposes such as in orthopaedic and in ear-nose-throat specialist cases. Importantly, our purpose was not to replace the MNI or ICBM templates for brain-only applications but to provide a big FOV template for applications such as source imaging and head modeling.

Typically, T1w imaging data is used to generate templates as these images have a high spatial resolution and signal-to-noise ratio. However, it is also possible to generate a template from FLAIR images^9,10^, which we have done here in addition to the T1w template. It is useful to have multi-contrast templates as this allows the researcher to better analyze deep gray matter structures that may not be readily visible on T1w images. Furthermore, multispectral tissue segmentation can be achieved through the additional use of a 3D FLAIR image that can improve the separation of gray matter tissue from pial, vessels and extra-cerebral connective tissue at brain edges^11,12^. In patients with negative conventional MRI and focal epilepsy, multispectral voxel-based morphometry (VBM), especially T1w + FLAIR, can yield superior results over single-channel (i.e. single modality) T1w segmentation^13^. We have therefore employed multispectral segmentations to generate our T1w and FLAIR templates. To our knowledge, this is the first T1w + FLAIR head and brain template with a big FOV.

## Methods

### Datasets

We used 225 control datasets from six studies. We complied with all relevant ethical regulations and informed consent was obtained from all subjects. The local ethics committees approved of each study. The ethics reference numbers were as follows: 646/2011BO1 for study 1, 115/2013BO2 for study 2, 295/2015BO1 for study 3, 390/2014B01 for study 4, 16/10/17 for study 5 and 2/5/21 for study 6. There was no history of psychiatric or neurologic diseases. Inclusion criteria were: (i) presence of a 3D T1w (MPRAGE, Magnetization Prepared Rapid Gradient Echo) and (ii) 3D FLAIR (T2-SPACE) image with a spatial resolution of a maximum of 1 mm^3^ for both modalities. All images were acquired on Siemens Healthcare (Erlangen, Germany) scanners. These included Prisma, Skyra, Prisma^fit^ and Biograph mMR scanners. All details are specified in Table 1.

**Table 1:** Breakdown of all study data, demographics and acquisition parameters.

Project	Site	Model	# Subjects	Age in years (mean ± STD)	Sex (m/f)	Scan	Echo Time (ms)	Repetition Time (ms)	Flip Angle (°)	Inversion Time (ms)	Resolution (mm^3^)	Matrix	Coil
1	Tübingen	Prisma	37	33.5 ± 12.8	16/21	T1w	3.03	2300	8	1100	0.99 × 1 × 1	176 × 256 × 224	64
						FLAIR	388	5000	120	1800	0.99 × 1 × 1	176 × 256 × 256	64
2	Tübingen	Skyra	86	37.2 ± 14.2	38/48	T1w	2.32	2300	8	900	0.89 × 0.89 × 0.89	192 × 256 × 256	32
						FLAIR	387	5000	120	1800	0.89 × 0.89 × 0.89	192 × 256 × 256	32
3	Tübingen	Biograph mMR	18	32.3 ± 9.2	5/13	T1w	2.49	1900	9	900	1 × 0.45 × 0.45	192 × 512 × 512	20
						FLAIR	386	5000	120	1800	1 × 1 × 1	192 × 256 × 256	20
4	Tübingen	Prisma	32	27.7 ± 5	18/14	T1w	2.98	2300	9	900	0.99 × 1 × 1	176 × 256 × 240	64
						FLAIR	388	5000	120	1800	0.99 × 1 × 1	176 × 256 × 256	64
5	Göttingen	Prisma^fit^	26	31.8 ± 9.4	15/11	T1w	3.3	2250	9	900	0.99 × 1 × 1	176 × 256 × 256	64
						FLAIR	394	5000	120	1800	0.99 × 0.98 × 0.98	192 × 256 × 256	64
6	Göttingen	Prisma^fit^	26	25.8 ± 5.6	9/17	T1w	3.3	2250	9	900	0.99 × 1 × 1	176 × 256 × 256	64
						FLAIR	394	5000	120	1800	0.99 × 0.98 × 0.98	192 × 256 × 256	64
Total	—	—	225	32.9 ± 12	101/124		—	—	—	—	—		

T1w = T1-weighted MRI, FLAIR = fluid-attenuated inversion recovery, m = male, f = female, STD = standard deviation, ms = milliseconds, mm = millimeter.

The T1w and FLAIR data quality was visually reviewed using FSL^14,15^ ‘slicesdir’ and no participant had to be excluded.

### Preprocessing

**Fig. 1:**
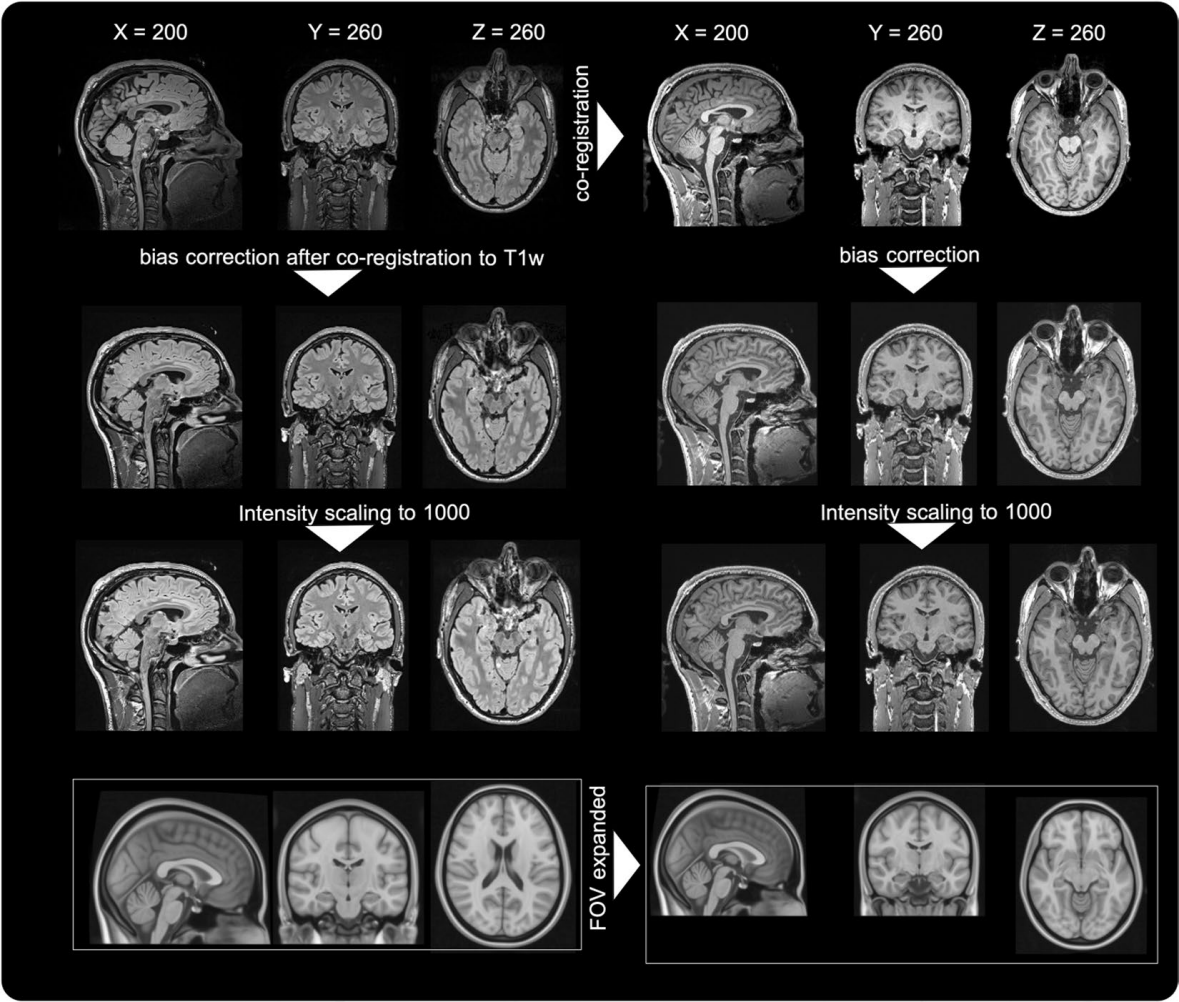
Preprocessing workflow. Images are in radiological convention (left in the image is right in the subject/template). MNI = Montreal Neurological Institute, FOV = field-of-view, T1w = T1-weighted image. FLAIR images were rigidly co-registered and resampled to their respective T1w counterparts per subject. Next, the brain was segmented into multiple tissue classes and we also performed registration and intensity non-uniformity (bias) correction. We standardized the image intensities for all images linearly setting the mean grey-matter intensity to 1000. Finally we expanded the FOV for the MNI template.

The entire preprocessing pipeline is shown in Fig. 1 and included the FLAIR to T1w coregistrations, the brain segmentation, bias correction and standardization through setting the mean grey-matter intensity to 1000 - all of these steps are subsequently described. To remove bias fields and allow intensity standardization we did a minimal preprocessing within MATLAB 2018b^16^ and SPM12^17^ (version 7487, http://www.fil.ion.ucl.ac.uk/spm/software/spm12, Fig. 1). FLAIR images were rigidly (6 degrees of freedom) co-registered and resampled to their respective T1w counterparts per subject using the “Coregister: estimate and reslice” option wihtin SPM. We have used the defaults, i.e. normalized mutual information and a 4th degree bspline interpolation algorithm was used for resampling. Next, a multispectral segmentation (“New Segment”^18^) was done. Here, the brain was segmented into multiple tissue classes such as gray and white matter and cerebral spinal fluid. This routine not only provides tissue segmentation, but also performs intensity non-uniformity (bias) correction, which is the only output used in this work. Resulting bias corrected T1w and FLAIR images were stored in native T1w-based space. SPM default settings were used in this process. Next, we normalized the image intensities for all images (T1w and FLAIR) linearly setting the mean gray matter intensity to 1000 (Fig. 1).

### Template generation

**Fig. 2:**
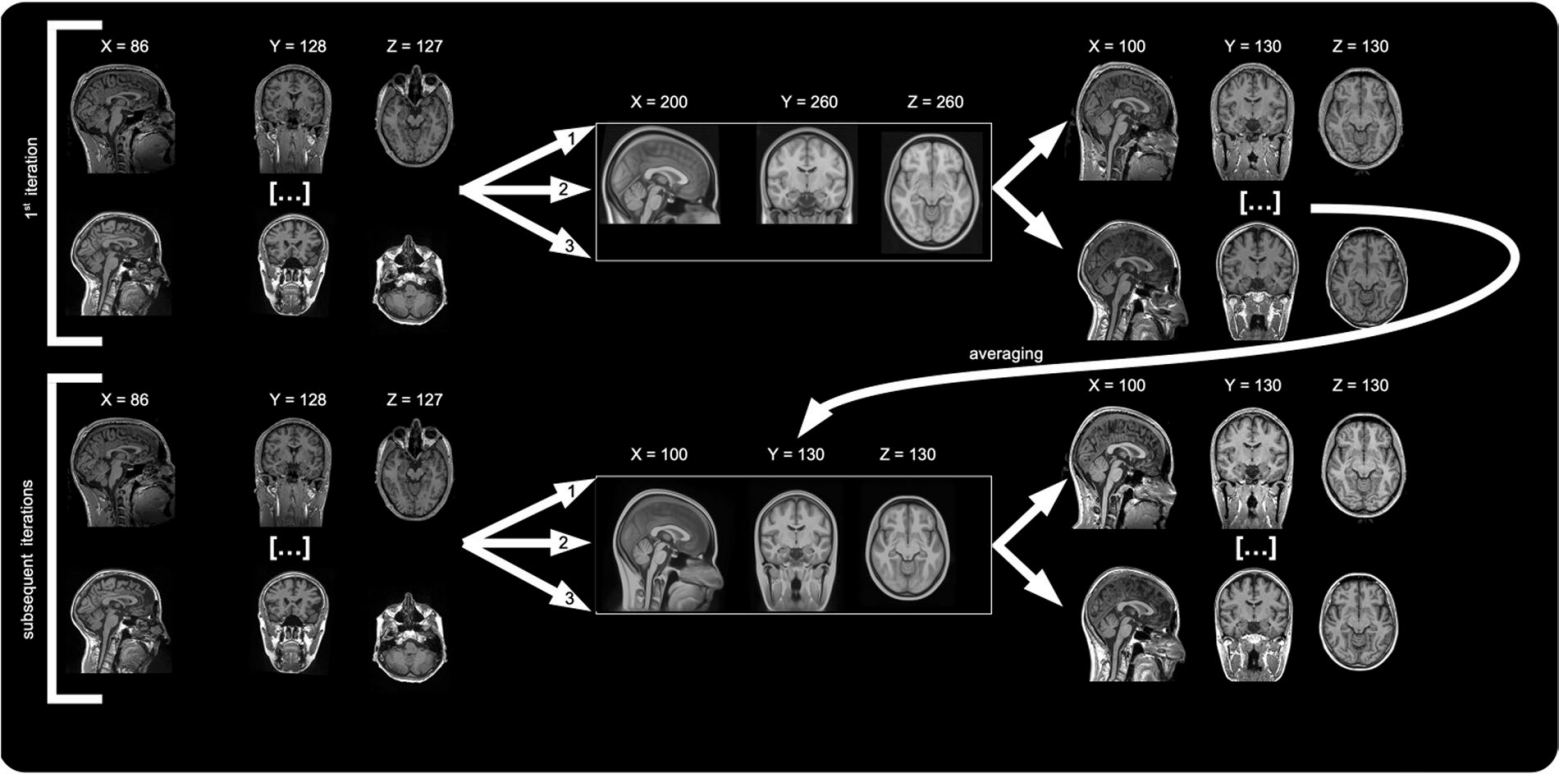
Template generation workflow. The process workflows for the first and subsequent iterations are shown, with the warped results on the right of this graph. The curved arrow indicates the averaging of all warped T1w images. 1 = 6 degrees of Freedom rigid-body alignment, 2 = affine registration, 3 = Syn registration. Images are in radiological convention (left in the image is right in the subject/template).

In Fig. 2, the template generation is depicted, showing the process workflows for the first and subsequent iterations. All template generation steps were performed within PYTHON (version 3.8; packages: gcc-9.3.0, nilearn 0.8.1, nibabel 3.2.1 and nipype version 1.7.1). As a first step, we expanded the FOV of the FSL MNI/MNI152 NLIN 6^th^ generation template using nibabel (Fig. 1). The isotropic 1 mm^3^ version was expanded from 182 × 218 × 182 voxels to 201 × 261 × 261 voxels, while the 0.5 mm^3^ version was expanded from 364 × 436 × 364 voxels to 402 × 522 × 522 voxels. Based on the big FOV MNI template, we also generated a binary FSL bet-based^19^ brain mask with BET defaults and a FOV-mask, i.e. a mask of original MNI152 FOV voxels in the expanded big FOV space. We then made sure that all T1w and FLAIR data had the same radiological orientation. Subsequently, we employed ANTs^20^ (http://stnava.github.io/ANTs/) with its standard three-step co-registration to a reference template, which in our case was the 1 mm big FOV file (Fig. 2). The first step was a rigid body registration (6 degrees of freedom) between each individual T1w image and the template. Then, the T1w image was registered to the template with an affine registration (12 degrees of freedom). SyN stands for symmetric normalization with affine and deformable transformations, with mutual information as optimization metric. Finally, this non-linear registration was performed using SyN (with settings for steps = 0.1, update variance penalty = 3, total variance penalty = 0) for the first through fifth iterations. The sixth and seventh iterations employed more SyN liberty at steps size of 0.2, update variance penalty = 1 and total variance penalty = 0. Other parameters are detailed in Table 2. This process was completed for each image and yielded 225 T1w images in template space and ANTs registration flows (.h5 files). Finally, the warped images were averaged to form a new template.

**Table 2:** Other co-registration and SyN parameters in ANTs. vox = voxels.

Name	Setting
interpolation	Bspline
use histogram matching	True
winsorize-image-intensities	[0.005,0.995]
transform	Rigid [0.1]
metric convergence shrink-factors smoothing-sigmas	[metricWeight = 1, numberOfBins = 32, samplingStrategy = Regular, samplingPercentage = 0.25] [1000 × 500 × 250 × 100,1e-6,10] 8 × 4 × 2 × 1 3 × 2 × 1 × 0 vox
transform	Affine[0.1]
metric convergence shrink-factors smoothing-sigmas	[metricWeight = 1, numberOfBins = 32, samplingStrategy = Regular, samplingPercentage = 0.25] [1000 × 500 × 250 × 100,1e-6,10] 8 × 4 × 2 × 1 3 × 2 × 1 × 0 vox
transform	SyN
metric convergence shrink-factors smoothing-sigmas	[metricWeight = 1, numberOfBins = 4] [100 × 70 × 50 × 20,1e-6,10] 8 × 4 × 2 × 1 3 × 2 × 1 × 0 vox

**Fig. 3:**
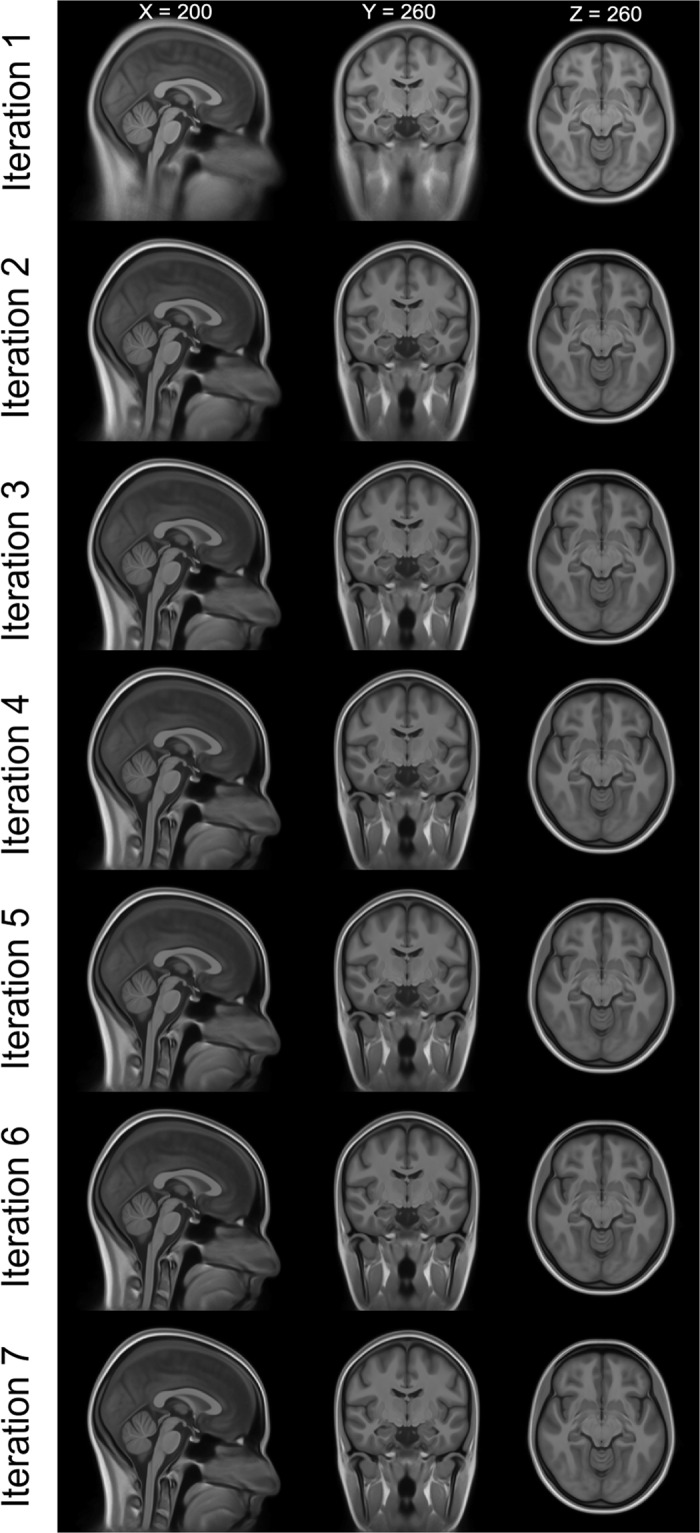
Results of the Iterations on the T1w template. Note how the extracranial areas gain in anatomical precision with the non-linear warping converging towards the higher iterations. After seven iterations the stop conditions of a root-mean-square error of less than 5% was reached. Images are in radiological convention (left in the image is right in the subject/template).

**Fig. 4:**
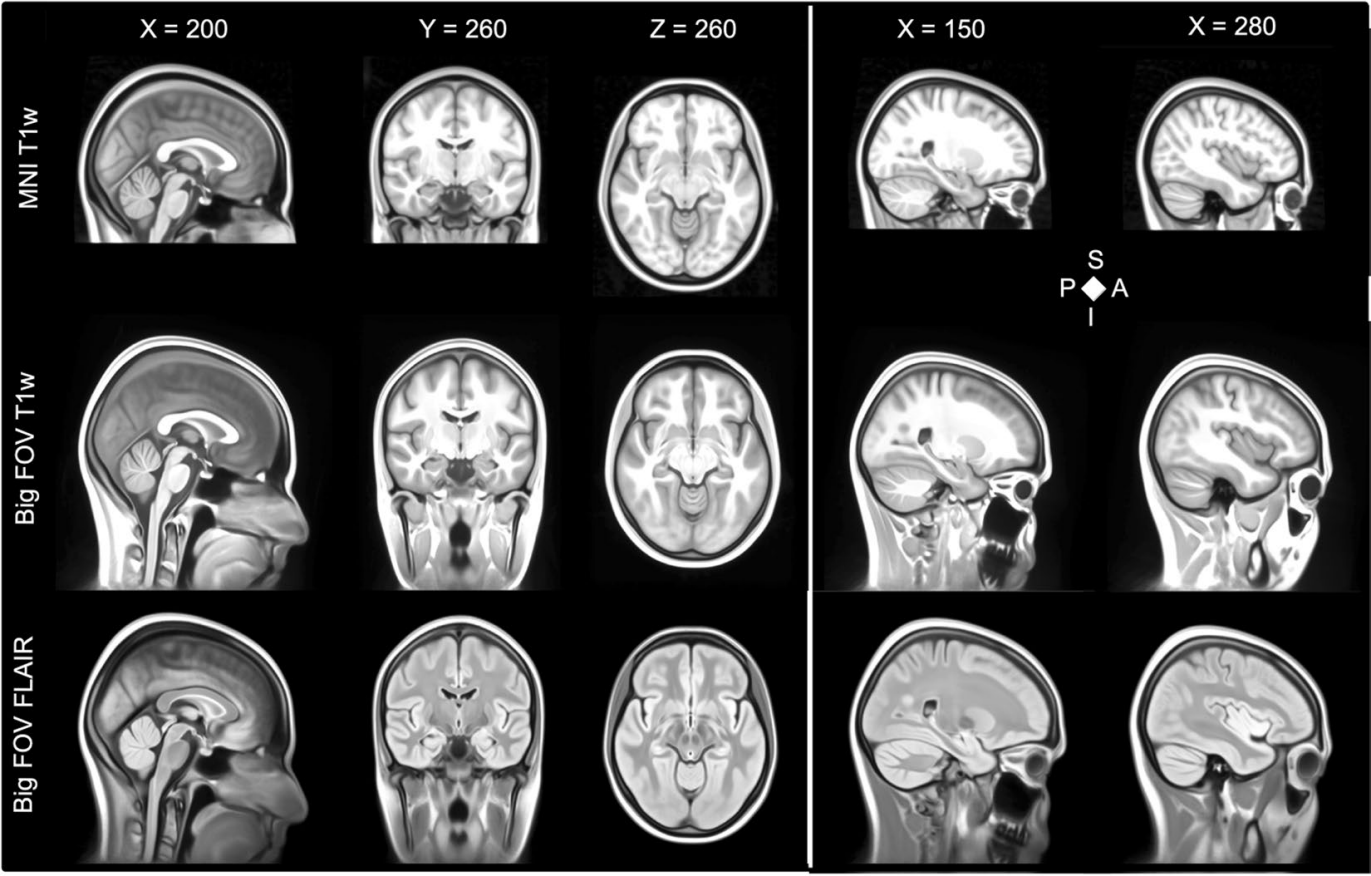
Generated full FOV templates of T1w and FLAIR in 0.5 mm^3^ resolution in comparison to the MNI T1w template. S = superior, P = posterior, I = inferior, A = anterior, T1w = T1-weighted image, MNI = Montreal Neurological Institute, FOV = field-of-view, FLAIR = fluid-attenuated inversion recovery. Images are in radiological convention (left in the image is right in the subject/template).

This process was done repeatedly in all iterations. In the first iteration, we used the expanded MNI152 template as registration target. Individual MR images were registered and warped to the initial big FOV template and an average was calculated. We then registered the resulting averaged iteration’s template again to the original MNI template to improve overall comparability. For this, we used the rigid, affine and Syn registrations. Only for the last/final iteration this process was skipped to maintain full template resolution and avoid interpolation effects. We constrained the affine registration estimation to the MNI152 original voxels (via the big FOV mask) and the non-linear registration to the MNI152 brain voxels (via the MNI152 brain mask). Results of the iterations on the T1w template are depicted in Fig. 3. We needed seven iterations (Fig. 3) to arrive at our average template (Fig. 4). The stop criterion for both levels of SyN liberty was a root-mean-square image intensity difference of below 5% between the template of the previous iteration and the current template. After the final iteration, we generated an average template of T1w and FLAIR in 1 mm^3^ and 0.5 mm^3^ resolution. The individual images were resampled with the existing transformations estimated based on T1w images. Figure 4 shows the generated full FOV templates of T1w and FLAIR in 0.5 mm^3^ resolution in comparison to the MNI T1w template.

Furthermore, we generated a template from a subset of the most representative subjects. For this, we used a more advance averaging method where only the twenty most representative subjects were averaged (i.e. those with the lowest cost function sum). We also provide this template at the GRO-link^21^.

## Data Records

We provide templates for every iteration^22^ (Fig. 2). The T1w and FLAIR templates are provided in NIfTI format at 1 mm^3^ and 0.5 mm^3^ (Fig. 4) isotropic resolution (NMRI225_T1.nii, NMRI225_T1_0.5 mm.nii, NMRI225_Flair.nii, NMRI225_Flair_0.5 mm.nii)^21^. The FLAIR and T1w templates are saved as float datatypes. We cannot make the original T1w data of all 225 participants available as these have not been defaced and therefore would not be anonymized.

## Technical Validation

Because we wanted to have a representative average template, we used a method to detect outliers based on previous work^23^. In brief, we calculated the default mutual information cost-functions of all image pairs (template registration target and co-registered images). Outliers per this definition were those images that had a cost function sum that was two interquartile ranges away from the median. These outliers were excluded from the template generation. In iterations one through five we found one outlier each. In the sixth and final iteration we had no more outliers, hence, all subjects were used in the final template generation.

**Fig. 5:**
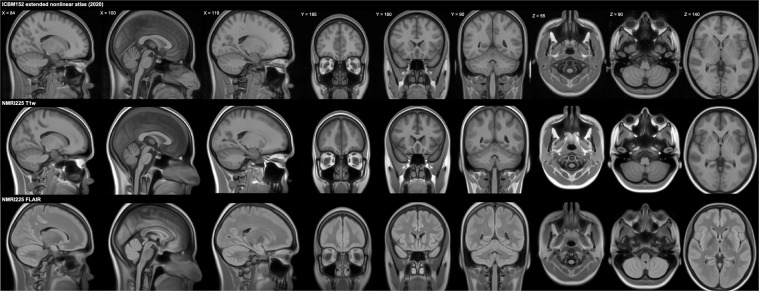
Comparison of NMRI225 and ICBM152 extended nonlinear atlas (2020) templates. Images are in radiological convention (left in the image is right in the subject/template).

Some cortical gray matter regions appear to be blurrier in our template than in the initial MNI 152 NLIN 6^th^ generation template. This is likely due to the different approach used in our work that is not intended to replace the MNI template(s) but to provide a larger field-of-view template that has high image quality also for extracranial areas. Image quality in extracranial and subcortical regions was improved at the cost of blurrier and less detailed cortical gray matter regions. The NMRI225 template should be preferred over the MNI 152 NLIN 6^th^ generation template for use cases where a big field-of-view with both T1w and FLAIR contrast is needed. In Fig. 5 we provide a comparison of our NMRI225 and the ICBM152 extended nonlinear atlas (2020) templates.

The compatibility to the existing MNI 152 6^th^ generation template was maximized but the shape and location of some brain structures in the proposed NMI225 template do slightly deviate from the existing MNI 152 6^th^ generation template because the registrations were performed without brain masks to improve the quality in extracranial regions. Since the main goal of this project was to generate a template including extra-cranial tissues using skull-stripped images would be counterproductive. Hence, this choice was done by design.

## Usage Notes

The templates can be generated using the researchers’ own datasets. We provided NMRI225_run.m and NMRI225_run.py, that should be run in this order.

## Data Availability

We make our code available at https://github.com/barbrakr/NMRI225.git as NMRI225_run.m, NMRI225_run.py and nmri_functions, under a CC BY license. We used MATLAB 2018b to run NMRI225_run.m and Python 3.8 for running NMRI225_run.py. We have summarized the packages of the conda repository in Supplementary Materials.
